# Impact of concomitant aortic regurgitation on long-term outcome after surgical aortic valve replacement in patients with severe aortic stenosis

**DOI:** 10.1186/1749-8090-6-51

**Published:** 2011-04-13

**Authors:** Suad Catovic, Zoran B Popovic, Nebojsa Tasic, Dusko Nezic, Predrag Milojevic, Bosko Djukanovic, Sinisa Gradinac, Lazar Angelkov, Petar Otasevic

**Affiliations:** 1 General Hospital, Novi Pazar, Serbia; 2 Cleveland Clinic, Cleveland, USA; 3 Dedinje Cardiovascular Institute and Belgrade University School of Medicine, Belgrade, Serbia

**Keywords:** aortic stenosis, aortic regurgitation, aortic valve replacement, long term outcome

## Abstract

**Background:**

Prognostic value of concomitant aprtic regurgitation (AR) in patients operated for severe aortic stenosis (AS) is not clarified. The aim of this study was to prospectively examine the impact of presence and severity of concomitant AR in patients operated for severe AS on long-term functional capacity, left ventricular (LV) function and mortality.

**Methods:**

Study group consisted of 110 consecutive patients operated due to severe AS. The patients were divided into AS group (56 patients with AS without AR or with mild AR) and AS+AR group (54 patients with AS and moderate, severe or very severe AR). Follow-up included clinical examination, six minutes walk test (6MWT) and echocardiography 12 and 104 months after AVR.

**Results:**

Patients in AS group had lower LV volume indices throughout the study than patients in AS+AR group. Patients in AS group did not have postoperative decrease in LV volume indices, whereas patients in AS+AR group experienced decrease in LV volume indices at 12 and 104 months. Unlike LV volume indices, LV mass index was significantly lower in both groups after 12 and 104 months as compared to preoperative values. Mean LVEF remained unchanged in both groups throughout the study. NYHA class was improved in both groups at 12 months, but at 104 months remained improved only in patients with AS. On the other hand, distance covered during 6MWT was longer at 104 months as compared to 12 months only in AS+AR group (p = 0,013), but patients in AS group walked longer at 12 months than patients in AS+AR group (p = 0,002). There were 30 deaths during study period, of which 13 (10 due to cardiovascular causes) in AS group and 17 (12 due to cardiovascular causes) in AS+AR group. Kaplan-Meier analysis showed that the survival probability was similar between the groups. Multivariate analysis identified diabetes mellitus (beta 1.78, p = 0.038) and LVEF < 45% (beta 1.92, p = 0.049) as the only independent predictor of long-term mortality.

**Conclusion:**

Our data indicate that the preoperative presence and severity of concomitant AR has no influence on long-term postoperative outcome, LV function and functional capacity in patients undergoing AVR for severe AS.

## Introduction

In routine clinical practice significant number of the patients with aortic stenosis (AS) have concomitant aortic regurgitation (AR) of different severity, which is easily explained having in mind etiology and pathological process responsible for development of stenosis of effective aortic valve orifice.

According to actual guidelines for treatment of patients with valvular heart diseases, in symptomatic patients with confirmed AS, surgical aortic valve replacement (AVR) is recommended, and the same approach is advised in the case of combined aortic valve disease, if the stenosis is predominant lesion [1].

Following successful AVR due to AS, in the majority of the patients significant symptomatic and functional improvement is noted, with significantly better long-term survival as compared to medically treated patients [2]. Factors that may influence outcome following AVR include age, preoperative NYHA class, left ventricular (LV) hypertrophy and ejection fraction (EF), heart rhythm disturbances, preoperative pressure gradient over aortic valve, and presence of coronary artery disease [3,4].

Prognostic value of concomitant AR in patients operated for severe AS is not clarified. Some investigators identified preoperative presence of significant AR as a risk factor for development of postoperative LV dysfunction, while others did not [5,6]. Impact of associated AR on long-term survival following AVR is also controversial [3,7].

Therefore, the aim of the present study was to prospectively examine the impact of presence and severity of concomitant AR in patients operated for severe AS on long-term functional capacity, left ventricular (LV) function and mortality.

## Methods

### Patients

The study population consisted of consecutive symptomatic patients with significant AS operated at Dedinje Cardiovascular Institute from January 1 to December 31, 1999. The study was prospective. Inclusion criteria included 1) mean gradient over aortic valve > 30 mmHg, 2) elective operation, 3) willingnes to sign informed consent. Exclusion criteria were 1) significant valvular disease, other than aortic valve, requiring surgery, 2) significant AR and associated AS with mean gradient over aortic valve ≤ 30 mmHg, 3) previous coronary and/or valvular surgery. Presence of significant coronary artery disease was not considered as exclusion factor.

A total of 110 patients met inclusion/exclusion criteria, and were included in the study. Patients were divided in two groups: AS group - patients with isolated symptomatic AS and significant AS with trace or mild (1+) AR; and AS+AR group - patients with significant AS and moderate (2+), severe (3+) or very severe (4+) AR. Their medical records were reviewed for demographic, clinical, and ECG data.

### Preoperative echocardiographic findings

Preoperative transthoracic echocardiographic assessment included standard M mode, 2D and color Doppler study using Sonos 2500 system (Hewlett Packard, Andover, MA, USA). LV volumes and EF were calculated from apical two- and four- chamber cross sections by using Simpson`s method. The apical five-chamber and/or suprasternal cross sections were used to obtain continuous wave Doppler recordings to measure maximal velocity across the aortic valve. Maximal systolic pressure gradient over aortic valve was calculated from the Doppler velocities using the modified Bernoulli equation. Aortic regurgitation was semiquantitatively assessed by Color Doppler flow, using standard technique. The LV mass (LVM) was calculated using the Devereux and associates equation as: LVM = 1,04 (LVEDD + IVSTd + PWTd)^3 ^× 0,8 + 0,6; where EDD = end-diastolic dimension, IVSTd = interventricular septal thickness at end-diastole and PWTd = posterior wall thickness at end-diastole, and corrected by ASE - cube conversion. Left ventricle mass and volumes were adjusted to body surface area and expressed as indexes. LV systolic dysfunction was defined as LVEF < 45%.

### Preoperative hemodynamic and angiographic findings

Preoperative invasive diagnostic included standard left cardiac catheterization with aortic root and coronary artery angiography in all patients. Pressure gradient was measured directly and aortic regurgitation was semiquantified as 0, 1+, 2+, 3+ i 4+. Coronary artery disease was defined as ≥ 50% lumen diameter narrowing of the left main coronary artery or ≥ 70% lumen diameter narrowing of at least one of the major epicardial vessels. Multivessel coronary artery disease was defined as either left main or two or three major epicardial vessel disease. In the case of disagreement with the estimation of the aortic regurgitation between echocardiographic and angiographic assessment, angiographic result was used for further analysis.

### Operative data

Aortic valve replacements were done by standard surgical procedure with cardiopulmonary bypass and cardioplegia. Mechanical prosthesis was implanted to all of the patients. Most often used prosthesis was Medtronic Hall, and rarely St. Jude or Carbomedicis. If indicated, concomitant coronary artery bypass surgery was performed at the same time as AVR, using standard technique. All surgeries were performed by 11 staff cardiac surgeons, and the details of preoperative patient management were left to the discretion of the attending physician.

### Follow-up

The first control examination was done 12 ± 3 months following surgery with 101 patients (1 patient lost to follow-up). Two patients died during the first postoperative year, and the data for time and cause of death were reviewed from the relevant medical documentation, supplied by patient's families. Examination included clinical assessment, ECG, echocardiography and six minute walk test (6MWT). The test was performed per protocol of Lipkin and associates [8], with encouragement during the test. Three patients were not willing to cooperate during the test and their results were excluded from further analysis.

The second control examination was done 104 ± 3 months after operation with 79 patients. A total of 22 patients who died during period between two follow up examinations, and the data for time and cause of death were reviewed from the relevant medical documentation, supplied by patient's families. The protocol was the same as for the first control examination. Echocardiography was performed by Vivid 4 system (General Electric, Milwaukee, WI, USA) for ehocardiographic assessment. Two patients were not willing to cooperate during 6MWT and their results were excluded from further analysis.

### Statistical analysis

Data are expressed as mean value ± standard deviation for continuous variables, and the paired and unpaired Student t-test was performed to determine intra- and intergroup differences between mean values. For categorical variables data are expressed as numbers with percentage, and were analyzed by chi-square test or Fisher's exact test, as appropriate. Predictors of long-term survival was tested using a univariate and multivariate analysis. Variables with p < 0,1 in univariate were included in multivariate analysis. A p < 0,05 in multivariate analysis was considered statistically significant. Survival was estimated by the use of Kaplan - Meier method, and a difference between survival curves was tested with a long- rank test. All statistics were processed by a standard statistical software package (SPSS release 10, SSPS Inc., Chicago, IL, USA).

## Results

### Preoperative and operative characteristics

Preoperative patient`s characteristics are presented in Table 1. Briefly, patients in AS group were significantly older and had more frequently hypertension. Patients in AS+AR group had significantly higher mean left ventricular end-diastolic volume index (EDVi), mean left ventricular end-systolic volume index (ESVi) and left ventricular mass index (LVMi). There were no differences between the group with respect to other preoperative variables.

**Table 1:** Preoperative demographic, clinical, echocardiographic, angiographic and haemodynamic data

	overall	AS	AS+AR	p
Number of patients	110	56	54	
Age (years) mean ± S.D.	60,5 ± 9,4	64,2 ± 5,64	56,7 ± 11,0	0,00003
Sex (n,% female)	33 (30)	21 (38)	12 (22)	0,0805
NYHA class mean ± S.D.	2,39 ± 0,49	2,34 ± 0,48	2,44 ± 0,50	0,7207
Symptoms (months) mean ± S.D.	18,1 ± 15,9	20,2 ± 16,8	15,9 ± 14,6	0,1599
Hypertension n,(%)	39 (35)	29 (52)	10 (18)	0,0003
Diabetes mellitus n,(%)	7 (10)	6 (11)	1 (2)	0,0569
Hiperlipoproteinemia n,(%)	23 (41)	15 (27)	8 (14)	0,1227
Bicuspid aortic valve n,(%)	33 (30)	11 (20)	20 (37)	0,0426
Atrial fibrilation n,(%)	4 (4)	3 (5)	1 (2)	0,3262
LV EDVi (ml/m^2^) mean ± S.D.	81,7 ± 21,2	71,3 ± 16,0	92,6 ± 29,2	0,0001
LV ESVi (ml/m²) mean ± S.D.	35,1 ± 15,4	28,9 ± 13,7	41,5 ± 22,9	0,0008
LV EF (%) mean ± S.D.	59 ± 14	60 ± 13	57 ± 15	0,2192
LV EF < 45% n,(%)	18 (16)	7 (13)	11 (20)	0,2646
LVMi (g/m²) mean ± S.D.	112,3 ± 20,5	106,9 ± 19,0	117,93 ± 20,6	0,0046
ΔP eho max (mmHg) mean ± S.D.	98 ± 29	98 ± 22	98 ± 35	0,9894
ΔP eho mean (mmHg) mean ± S.D.	63 ± 19	63 ± 16	62 ± 22	0,9393
Coronary artery disease n,(%)	29 (26)	18 (32)	11 (20)	0,1454
ΔP cath (mmHg) mean ± S.D.	85,3 ± 28,3	89,7 ± 25,8	81,1 ± 30,3	0,2455

*Abbreviations: LV - left ventricle, EDVi -end-diastolic volume index, ESVi -end-systolic volume index, EF - ejection fraction, LVMi -left ventricular mass index, DP -pressure gradient, eho - echocardiografic, cath - catheterization, S.D. - standard deviation*.

The total operative mortality was 5% (6/110 patients). The operative mortality was similar in AS and AS+AR group (1.8% vs 9.2%, respectively, p = 0,084). Additionally, there were no differences between the groups with respect to other operative characteristics (Table 2).

**Table 2:** Operative characteristics

	overall	AS	AS+AR	p
Prosthesis type n, (%)				
Medtronic Hall	96 (87)	50 (90)	46 (85)	0,5189
Carbomedicis	4 (4)	1 (2)	3 (6)	0,2998
St.Jude	10 (9)	5 (9)	5 (9)	0,9519
Prosthesis size (mm)	22,62 ± 1,90	22,13 ± 1,83	23,13 ± 1,85	0,9231
Prosthesis size/BSA (mm/m²)	12,20 ± 1,21	12,19 ± 1,23	12,20 ± 1,19	0,9531
Bypass surgery n, (%)	22 (20)	12 (21)	10 (19)	0,7029
Single bypass	4 (4)	2 (3)	2 (4)	0,3886
Double bypass	12 (11)	5 (9)	7 (13)	0,4974
Triple bypass	6 (5)	5 (9)	1 (2)	0,1023
Operative mortality n, (%)	6 (5)	1 (2)	5 (9)	0,0844

Abbreviations: BSA - body surface area

### Follow-up data

**Figure 1:**
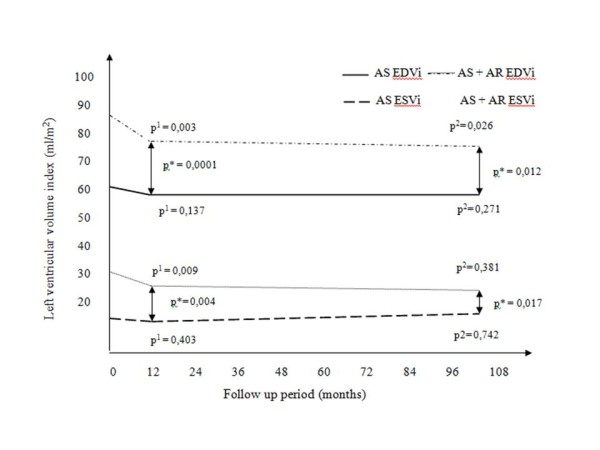
**Change of mean LV end-diastolic and end-systolic volume indexes during follow up period**. Abbreviations: AR, aortic regurgitation; AS, aortic stenosis; EDVi, end-diastolic volume index, ESVi, end-systolic volume index; LV, left ventricle. P* marks difference between groups, p^1 ^marks difference between preoperative values and values on the first control, p^2 ^marks difference between preoperative values and values on the second control

**Figure 2:**
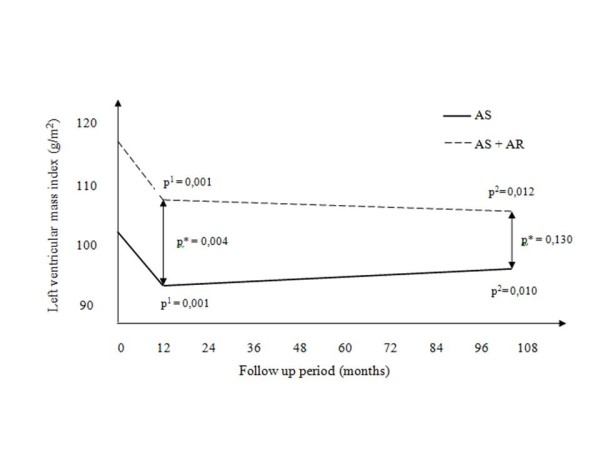
**Change of LV mass index during during follow up period**. For abbreviations and details see Figure 1.

Changes in LVEDVi and LVESVi during follow-up period are shown on Figure 1. It can be appreciated that the patients in AS group had lower LV volume indices throughout the study than patients in AS+AR group. On the other hand, patients in AS group did not have postoperative decrease in LV volume indices, whereas patients in AS+AR group experienced decrease in LV volume indices at 12 months, which was evident also after 8 years postoperatively at 104 months. Figure 2 depicts changes in LVMi during the study. Unlike LV volume indices, LVMi was significantly lower in both groups after 12 and 104 months as compared to preoperative values. Additionally, LVMi was lower preoperatively and 12 months after AVR in patients with AS alone in comparison with patients with AS+AR, but at 104 months LVMi was similar between the groups. Mean LVEF remained unchanged in both groups throughout the study, as well as the number of patients with depressed LVEF (predefined as <45%) (Table 3).

**Table 3:** Results on control examinations according to defined outcomes

Outcome	Group	Preoperatively	First control	Second control
NYHA class mean ± s.d.	AS	2,34 ± 0,48	1,98 ± 0,42^1^	2,07 ± 0,46^1^
	AS + AR	2,44 ± 0,50	2,08 ± 0,45^1^	2,22 ± 0,58
6MWT (m) mean ± s.d.	AS	nd	322 ± 96	340 ± 100
	AS + AR	nd	276 ± 106^3^	325 ± 89^2^
LV EF (%) mean ± s.d.	AS	60 ± 13	62 ± 11	60 ± 10
	AS + AR	57 ± 15	60 ± 11	57 ± 11
LV EF < 45% n, (%)	AS	7 (13)	3 (8)	2 (6)
	AS + AR	11 (20)	3 (9)	4 (12)
Dead n, (%)	AS	-	1 (2)	12 (22)
	AS + AR	-	1 (2)	12 (24)

*Abbreviations: *nd, not done; ^1 ^p < 0.001 vs preoperative values; ^2 ^p < 0.05 vs AS group; ^3 ^p < 0.05 vs first control

As shown in Table 3, NYHA class was improved in both groups at 12 months, but at 104 months remained improved only in patients with AS. On the other hand, distance covered during 6MWT was longer at 104 months as compared to 12 months only in AS+AR group (p = 0,013), but patients in AS group walked longer at 12 months than patients in AS+AR group (p = 0,002).

**Figure 3:**
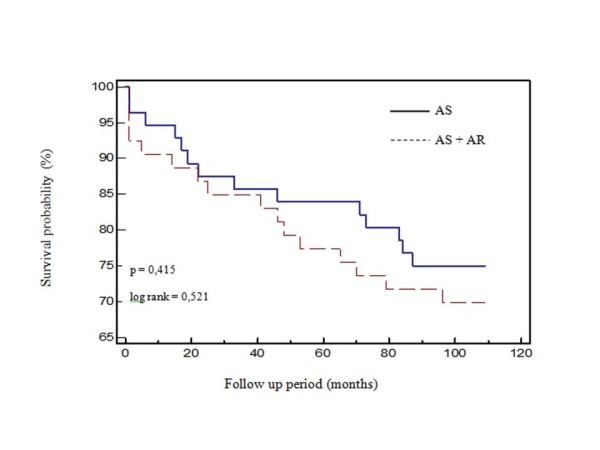
**Kaplan - Meier survival curves following surgery; a comparison of the patients with preoperative isolated aortic stenosis versus aortic stenosis with associated significant aortic regurgitation For abbreviations see Figure 1**.

During the course of the study only 1 patient was lost to follow-up (0.9%). There were 30 deaths, of which 13 (10 due to cardiovascular causes) in AS group and 17 (12 due to cardiovascular causes) in AS+AR group. Kaplan-Meier analysis showed that the survival probability was similar between the groups (Figure 3).

In order to assess prognostic impact of preoperative demographic, clinical, echocardiographic and angiographic variables, we tested a number of these variables in univariate model (age, sex, NYHA class, symptoms duration, hypertension, diabetes mellitus, hyperlipoproteinemia, atrial fibrillation, presence and severity of associated aortic regurgitation, LV volume indices, LVEF, LV systolic dysfunction (LVEF < 45%), LV mass index, maximal and mean pressure gradient over aortic valve, presence of coronary artery disease). Of the tested variables, only diabetes mellitus (beta 1.62, p = 0.044), preoperative LVEF (beta -1.19, p = 0.063) and LVEF < 45% (beta 2.23, p = 0.015) emerged as univarite predictors of long-term mortality and were entered in multivariate model. Multivariate analysis identified diabetes mellitus (beta 1.78, p = 0.038) and LVEF < 45% (beta 1.92, p = 0.049) as the only independent predictor of long-term mortality.

### Discussion

Our data indicate that the preoperative presence and severity of concomitant AR has no influence on long-term postoperative outcome, LV function and functional capacity in patients undergoing AVR for severe AS.

Preoperative characteristics of both groups in our study were similar except for the age (AS group older) and LV volume indices (higher in AS+AR group). The reason for the discrepancy in age is probably the fact that AS in older patients is most commonly consequence of degenerative process with calcification of the valve leaflets [9], while in younger patients it is mostly due to congenital aortic valve diseases [9]. Additionally, coexisting AR is more frequent in younger patients [9], which is similar to the findings of our study. Significantly higher preoperative LV volumes and pronounced LV hypertrophy in patients with AS and coexisting significant AR, in relation to patients with isolated AS, was also noted in earlier reports [5,6].

There is ongoing controversy with respect to the impact of perioperative AR on long-term outcome following AVR due to severe AS. For the problem to be worse, it is difficult to make direct comparisons of different studies due to differences in the methodology. For example, some studies who examined outcome after AVR for AS included only patients with associated mild AR, [3,6], whereas other studies included patients regardless of the degree of associated AR [10]. Some authors separately analyzed patients with pure AS and patients with AS and mild or moderate, [11] while other authors analyzed only patients with AS and moderate or severe AR [5]. Previous studies identified sex, pressure gradient over aortic valve, type and size of the implanted prosthesis, and the incidence of associated coronary artery bypass surgery as a predictors of long term outcome of surgery [3,4,12,13]. Our data suggest that there is no difference in perioperative mortality between the AS and AS+AR groups, which is similar to previously reported paper [14].

Additionally, univariate and mulivariate analysis in our study failed to identify associated aortic regurgitation as a risk factor for long-term survival. The fact that univarite predictors of long-term mortality were diabetes mellitus, preoperative LVEF and LVEF < 45%, as well as that only diabetes mellitus and LVEF < 45% were identified as the only independent predictor of long-term mortality, are in concordance with previous studies [3].

The process of LV remodeling after AVR, in the sense of reduction of volumes, is most intense during first postoperative year [10,15,16], which is consistent with our findnigs. Despite pronounced reduction of LV volumes in AS+AR group, they were significantly higher than in AS group on both of the follow up examinations. This is in accordance to findings of other authors who followed patients with similar characteristics [5,6]. Although evident difference in LV volume indices was noted between the groups, there was no difference in LVEF and the number of patients with impaired LV function. Therefore, it can be postulated that in patients with AS and appropriate preoperative LV adaptation, capable to preserve LV systolic function, postoperative LV function will also be preserved regardless to the degree of coexisting AR. In other words, in patient with AS, if preoperative LV adaptation is appropriate, similar long term outcome according to LV systolic function can be expected, regardless to the type of preoperative adaptation. Also, it is well known that AVR due to AS has favorable impact on LV function and survival in the patients with reduced LV function [17,18].

In our study, postoperative LVMi was decreased in relation to preoperative values on both of follow up examinations, in both of the groups. This was not surprising, as regression of LV hypertrophy after surgery due to AS was previously confirmed by many investigators [19-21]. One year after AVR mean LVMi was significantly higher in AS+AR group, but at 104 months there was no difference between groups according to LVMi, which is similar to findings of Waszyrowski with associates [15]. Obviously LV readaptation following AVR, in patients with isolated AS and AS with concomitant AR, has different time course [6,21].

In the majority of patients AVR due to AS is followed by significant symptomatic improvement [5,17,18], where personal perception of improvement of the exercise tolerance was achieved mostly during the first postoperative year. Gradual, albeight non-statistically significant, increase in NYHA class in both groups in our study between two control examinations is most likely due to the fact that there was almost 8 years gap between the examinations, and that patients got older which might change personal perception of their exercise tolerance.

Objective measures of functional capacity, such as 6MWT, are rarely performed in follow-up of patients with AVR due to severe AS. It is well known that in heart failure patients 6MWT can identify patients with increased risk of mortality and morbidity [22,23]. are showed prognostic value of 6MWT in relation to survival in patients with heart failure. We have shown that distance covered during 6MWT was longer at 104 months as compared to 12 months only in AS+AR group, but patients in AS group walked longer at 12 months than patients in AS+AR group. The possible clinical importance of these data is not clear, but may reflect LV diastolic properties which were not assessed in this study. This issue is very important and merits further investigation in appropriately designed studies.

In this paper we showed favorable effect of AVR due to AS regarding long term survival, as it was confirmed in many other studies. In research of Craver and associates [9], in which the patients with AS and coexisting AR were observed jointly regardless of degree of associated AR, one year postoperative survival was 91% and 8-years survival was 76%. In research of Lund [3], in patients with AS and associated mild and moderate AR, 5-years postoperative survival was 85% and 10-years 68%. We did not find significant difference regarding long term survival between the groups, so it appears that preoperative presence of hemodinamically significant AR in patients with AS has no influence on long-term postoperative survival. This is a very controversial issue, since only one paper is in according with this finding, [3] while other authors identify associated AR as a risk factor for worse survival [7].

In conclusion, our data indicate that the preoperative presence and severity of concomitant AR has no influence on long-term postoperative outcome, LV function and functional capacity in patients undergoing AVR for severe AS.

## Authors' contributions

SC have made substantial contributions to conception and design, acquisition of data, analysis and interpretation of data; have been involved in drafting the manuscript and revising it critically for important intellectual content; have given final approval of the version to be published.

ZBP have made substantial contributions to conception and design, analysis and interpretation of data; have been revising manuscript critically for important intellectual content; have given final approval of the version to be published.

NT have made substantial contributions to conception and design, analysis and interpretation of data; have been revising manuscript critically for important intellectual content; have given final approval of the version to be published.

DN have made substantial contributions to analysis and interpretation of data; have given final approval of the version to be published.

PM have made substantial contributions to analysis and interpretation of data; have given final approval of the version to be published.

BD have made substantial contributions to analysis and interpretation of data; have given final approval of the version to be published.

SG have made substantial contributions to analysis and interpretation of data; have given final approval of the version to be published.

LA have made substantial contributions to analysis and interpretation of data; have given final approval of the version to be published.

PO have made substantial contributions to conception and design, analysis and interpretation of data; have been involved in drafting the manuscript and revising it critically for important intellectual content; have given final approval of the version to be published.
